# Public health concerns surrounding the cVDPV2 outbreak in Africa: Strategies for prevention and control with a special focus on Nigeria

**DOI:** 10.1002/hsr2.1269

**Published:** 2023-05-12

**Authors:** Ridwan O. Adesola, Ibrahim Idris, Emmanuel Opuni

**Affiliations:** ^1^ Department of Veterinary Medicine, Faculty of Veterinary Medicine University of Ibadan Ibadan Nigeria; ^2^ Department of Veterinary Medicine, Faculty of Veterinary Medicine Usmanu Danfodiyo University Sokoto Nigeria; ^3^ Department of Health Policy The London School of Economics and Political Science London UK

**Keywords:** Africa, cVDPV2, Nigeria, poliovirus

## Abstract

**Background and Aim:**

Poliovirus is a global health issue that affects children in different parts of the world. Despite the efforts of national, international, and nongovernmental organizations to eradicate the disease, it is re‐emerging in Africa due to poor sanitation, vaccine hesitancy, new ways of transmission, and poor surveillance among others. Circulating vaccine‐derived poliovirus type 2 (cVDPV2) is a major step in eradicating poliovirus and preventing outbreaks in developing countries. Strengthening African healthcare systems, increasing surveillance, hygiene and sanitation, and proper mass vaccination to achieve herd immunity are required in the fight against polio disease. This paper discusses the outbreak of cVDPV2, public health challenges, and recommendations in Africa with a special emphasis on Nigeria.

**Methods:**

We searched for articles documenting the incidence of cVDPV2 in Nigeria and other African countries on Pubmed, Google Scholar, and Scopus.

**Results:**

A total of 68 distinct cVDPV2 genetic emergences were found across 34 nations between April 2016 to December 2020, and in Nigeria, three cVDPV2 emergences were found. Also, 1596 instances of acute flaccid paralysis linked to cVDPV2 outbreaks were reported in four areas of the World Health Organization where Africa contributed 962 cases out of 1596 cases. Available data indicate that Africa has the most cVDPV2 cases and is associated with various challenges like the unidentified virus source, poor sanitation system, and inability to achieve herd immunity of the cVDPV2 vaccine.

**Conclusion:**

Collaborative efforts of stakeholders are crucial in combating infectious diseases, especially those transmitted via environments such as water and air, like poliovirus. Therefore, a collaboration between environmental health workers, veterinarians, community health workers, laboratory scientists, policymakers, and other professionals is required.

## 1 INTRODUCTION

Despite the tremendous effort by governments, the World Health Organization (WHO), United Nations International Children's Emergency Funds (UNICEF), Global Polio Eradication Initiative (GPEI) in the prevention of circulating vaccine‐derived poliovirus type 2 (cVDPV2) globally, outbreaks of these viruses have been occurring in a different part of the world. This is more evident in African countries that have indigent healthcare delivery services with poor sanitation and hygiene.^1^ cVDPV2 outbreaks is a great burden to GPEI because it is challenging to convince health professionals and the affected communities that administering the same vaccine with high coverage is the best method for controlling outbreaks and preventing re‐emergences.^2^

Between April and July 2022, a cVDPV2 with acute paralysis was identified in Algeria, Africa. The case was reported to World Health Organization by the Global Polio Laboratory Network. The issue was identified in a toddler, under two, from the Southern Algerian region of Tamanrasset who was diagnosed with acute flaccid paralysis. However, this is the first case ever found in the country. Following laboratory diagnosis of a fecal sample, the Pasteur Institute of Algeria and the Pasteur Institute of Paris revealed circulating vaccine‐derived poliovirus 2 in the sample. According to genomic sequencing analysis, the newly discovered virus was genetically related to the one previously discovered in Kano, Nigeria. However, the child had not gotten any doses of the polio vaccine and had never traveled outside the province of Tamanrasset.^3^ There is no evidence or information on how the child contracted the virus; *calling for* further studies focusing more on studying the mechanism of the virus transmission.^4^ Kano state is a metropolis in northern Nigeria with more than four million residing within a 449‐km square area; it is the second largest state in Nigeria after Lagos. Situated in the savannah, south of the Sahel, Kano state has attracted a large number of foreigners from different parts of the world, especially people from Asia and Africa, as it is a key trans‐Saharan trade route.^5^ This paper discusses the outbreak of cVDPV2, public health challenges, and recommendations in Africa with a special emphasis on Nigeria.

## 2 POLIOMYELITIS

Poliomyelitis is a viral infection caused by a virus belonging to the *picornaviridae* family. It is a very contagious infection with a milder episode of respiratory sickness, gastroenteritis, and malaise to severe types of paralysis. This has been categorized into in‐apparent infection, abortive poliomyelitis, nonparalytic poliomyelitis, and paralytic poliomyelitis is the disease's hallmarks.^6^ Poliovirus had a cosmopolitan distribution before the introduction of mass immunization campaigns, which may be due to poor environmental hygiene in low and middle countries. The highest incidence of the disease occurs in industrialized and temperate countries.^7^

Person‐to‐person contact is the main route of transmission of the virus. *Following* infection, the virus enters through the mouth and invades the intestine, which is subsequently expelled into the environment through the feces (feacal‐oral route of transmission).^2^ This virus further spread into the environment, particularly under poor hygiene and sanitation conditions.^2^ However, when a considerable number of children are protected against the virus, its infection rate will reduce because it cannot infect any more children who are susceptible or vulnerable to infection.^8^

Environmental surveillance is used to detect poliovirus circulation, especially in environmental sewage, and to track transmission in communities as part of the WHO's global action plan for eradicating poliomyelitis. Environmental surveillance supplements clinical acute flaccid paralysis surveillance for potential polio cases.^9^ It has been discovered that asymptomatic people shed significant amounts of the virus into the sewerage system.^10^ In developing countries, particularly Nigeria, in areas where water is scarce, farmers use water from sewages to water their plants—mostly vegetables and fruits—which are sold to people for consumption, making them susceptible to infections.

A total of 68 distinct cVDPV2 genetic emergences were found across 34 nations between April 2016 to December 2020, and in Nigeria, 3 cVDPV2 emergences were found. Also, 1596 instances of acute flaccid paralysis linked to cVDPV2 outbreaks were reported in four areas of the World Health Organization, where Africa has 962 cases out of 1596—12 cases in Nigeria, 5 cases in Angola, 3 cases in Chad, 1 case in Benin, 4 cases in Cameroon, 8 cases in the Central African Republic, 2 cases in Burkina Faso, 1 case in China, 2 cases in Cote d'Ivoire, 15 cases in the Democratic Republic of the Congo, 1 case in Guinea, 2 cases in Sudan, 9 cases in Ethiopia, 3 cases in Congo, 1 case in Ghana, 1 case in Liberia, 2 cases in Mali, 1 case in Mozambique, 3 cases in Somalia, 1 case in Egypt, 1 case in Niger, 1 case in Kenya, 1 case in Senegal, 1 case in Sierra Leone, and 1 case in South Sudan.^11^

## 3 CURRENT EFFORTS

Following the virus outbreak between April and July 2022, public health responses were put in place by the health authorities in Algeria and the World Health Organization to contain the virus's spread and prevent further outbreaks in susceptible populations and children with immunity against the virus. Some of the essential public health responses include.

### 3.1 Algeria

Surveillance was stepped up in the area surrounding the reported case to mop up any cases of acute paralysis. Again, to determine the extent of the viral circulation, a thorough field investigation was launched in conjunction with the Global Polio Laboratory Network, an organization aimed at differentiating acute flaccid paralysis caused by various diseases and the acute flaccid paralysis induced by the poliovirus. In compliance with the updated international polio outbreak response standard operating procedures,^12^ a response was activated. So also, a reactive vaccination effort was considered.

### 3.2 Nigeria

Interestingly, Nigeria continues to experience the transmission of vaccine‐derived poliovirus (cVDPV2) in spite of the wild poliovirus being declared eradicated in August 2020.^13^ In 18 states, including Kano, where four children have previously been proven to have the condition that causes paralysis, there have been new reports of the cVDPV2 being prevalent. The World Health Organization helped the Kano State administration to design and carry out a mass vaccination exercise with the cutting‐edge Oral Polio Vaccine Type 2, to stop the spread of the cVDPV2 epidemic. Four adjacent states also affected were synchronized with the outbreak response program. After the vaccination campaign on August 4, 2021, 3,297,347 infants and toddlers (ages 0–59 months), that is 99% of the Kano State children targeted for vaccination, had received their shots.^13^

### 3.3 Horn of Africa (Kenya, Somalia, and Ethiopia)

There were cases in different countries covering the horn of Africa. For Instance, Ethiopia's Somali province reported a cVDPV2 epidemic.^14^ This manifested acute flaccid paralysis with the disease that started on May 20, 2019. Subsequent genetic testing demonstrates that the isolated virus is connected to a cVDPV2 outbreak that was discovered in the Horn of Africa in 2018 and there have since been reported cases in both Somalia; and an environmental sample identified from Kenya.^14^ Ethiopia, together with Kenya and Somalia, declared the outbreak as a regional public health emergency and took part in the regional outbreak response since the cVDPVs was discovered in the Horn of Africa in 2018. Somalia is at a high risk of further spreading this isolated cVDPV2 due to their population's movements across borders in the horn of Africa, the subnational immunity, and surveillance gaps. Normally, the GPEI partners support when needed as the Ministry of Health and local health authorities conduct thorough investigations. Active surveillance was intensified, subnational population immunity levels were analyzed, and an epidemic response was developed; coupled with epidemiological and virological field study.

### 3.4 Sudan

On December 18, 2022, the International Health Regulations (IHR) National Focal Point of Sudan notified WHO of the detection of a cVDPV2 in a 48‐month‐old male from West Darfur in Western Sudan who had acute flaccid paralysis.^15^ This paralysis, is believed to have started on October 31, 2022. The isolated virus is unrelated to a cVDPV2 strain that caused an outbreak in Sudan in 2020 and it is most closely related to a variant that circulated in Borno State, Nigeria, in 2021. The Federal Ministry of Health began an immunization program for kids under 13 on November 28 in the impacted regions. With assistance from the GPEI's partners, local and national public health authorities quickly began conducting field investigations.^15^

## 4 GENERAL PUBLIC HEALTH CHALLENGES

The challenge associated with the current outbreak of cVDPV2 is the identification of the source of the virus. *Now*, the available clue is that the virus which is circulating around is genetically related to a previously identified virus in Kano and Borno State, Nigeria. If the source of the infection is identified first, it will provide insight into preventing the spread and transmission of the virus. Furthermore, Africa has been facing challenges and the burden of infectious diseases for an extended period; despite the efforts made in the prevention and controlling of infection, the burden of infectious diseases in African countries is probably associated with vulnerable healthcare facilities and poor hygiene and sanitation. Still, a major factor may be vaccine hesitancy, a situation where individuals do not accept vaccines due to their side effects and other reasons. However, this is mostly associated with a need for more education and enlightenment on the importance of vaccination and immunization. Another important challenge faced by Africa in the context of infectious diseases burden is vaccine equity; it is very important to ensure that everyone in the world has equal access to the vaccine.

### 4.1 Trackable recommendations for the affected countries

#### 4.1.1 Intensifying of surveillance

Surveillance is an essential tool in a disease investigation and identifying novel sources of infection and infectious agents. There is a need to increase surveillance in different regions of Nigeria, Algeria, and other neighboring countries. The Global Polio Laboratory Network should collaborate with other standard diagnostic laboratories in sequencing the virus, which will provide a full understanding of the genetic evolution of the virus.

#### 4.1.2 Vaccine equity and efficacy

Vaccine equity is achievable in Africa only if a tremendous effort is made by governments, nongovernmental organizations, health workers, and the entire community. Also, policymakers and community leaders have a role in ensuring that every individual accepts vaccines. At the same time, health professionals should educate and sensitize individuals on how important vaccination and immunizations are. Despite the monthly immunization against poliovirus, the virus keeps circulating. Therefore, tracking how the vaccine is transported, stored, and delivered is necessary to ensure that individuals receive an effective and quality vaccine. Training health workers on effective vaccine delivery is also essential.

*Strict monitoring of international travel:* international travel should be strictly monitored in airports, seaports, and neighboring countries' borders when travelers are moving or coming from endemic countries.

#### 4.1.3 Sensitization of the public

Governments and nongovernmental organizations should educate individuals on the effect of defecation in water bodies. This will help to combat poliovirus outbreaks that can be spread through environmental contamination. Therefore, governments should provide clean and safe water for the community.

#### 4.1.4 Adequate support from the developed Nations

The developed Nations in America, the United Kingdom, Europe, and Asia should keep assisting developing countries with vulnerable healthcare facilities with funds, diagnostic facilities, and vaccines against poliovirus and other infectious agents. Timely collaboration of the high‐income countries with African countries will help give the world health security.

#### 4.1.5 One health approach

One health is a collaborative, multisectoral, and transdisciplinary approach that operates at the local, regional, national, and global levels to achieve the best possible health outcomes by comprehending the links between people, animals, plants, and their environments.^16^ The need for utilizing this approach to the control of polio disease is important for achieving a polio‐free community in Africa. The system's efficiency will rise because of this integration, which will quicken decision‐making procedures and enhance coordination.

## 5 CONCLUSION

The Polio outbreak is re‐emerging despite the efforts of various actors including WHO, NGOs, governments, and health authorities. Poor sanitation, vaccine hesitancy, new ways of transmission, and poor surveillance are some of the contributory factors to the re‐emergence of the disease. To prevent the re‐emergence of the disease, surveillance should be intensified, efforts should be made toward vaccine equity, international travelers should be properly monitored, public involvement should be increased, One Health Approach should be adopted, and high‐income countries should continue to support Africa. A collaboration between environmental health workers, veterinarians, community health workers, laboratory scientists, policymakers, and other professionals at the national, regional, and district levels is crucial in fighting and preventing the re‐emergence of the disease, and for that matter polio‐free Africa.

## AUTHOR CONTRIBUTIONS

**Ridwan O. Adesola**: Conceptualization; data curation; resources; writing—original draft; writing—review and editing. **Ibrahim Idris**: Data curation; resources; writing—original draft; writing—review and editing. **Emmanuel Opuni**: Writing—original draft; writing—review and editing.

## CONFLICT OF INTEREST STATEMENT

The authors declare no conflict of interest.

## TRANSPARENCY STATEMENT

The lead author Ridwan O. Adesola affirms that this manuscript is an honest, accurate, and transparent account of the study being reported; that no important aspects of the study have been omitted; and that any discrepancies from the study as planned (and, if relevant, registered) have been explained.

## Data Availability

Not applicable.
